# Practical Departments

**Published:** 1901-10-05

**Authors:** 


### PRACTICAL DEPARTMENTS.

### INVALID FURNITURE.

(George Farmer and Co., Ltd., 12 Brompton Road London, S.W.)

Mr. George Farmer has opened some excellent show-rooms for invalid furniture at 12 Brompton Road. These we have visited, and amongst other useful commodities we noticed an inexpensive Ilkley couch, well made and nicely finished; this may be had in stained walnut, mahogany, or finished birch, polished. The centres are of cane; the couch can be inclined at any angle; there is an adjustable back and foot-rest, and, a great advantage for travelling, the legs can be screwed out for packing. Another very comfortable lounge has a most ingenious and simple folding and adjusting mechanism, without levers, springs, screws or complications of a kind calculated to irritate a nervous invalid. For use with these, or by the bed-side, are some very moderately-priced reading machines with telescopic stands so that the height can be altered when necessary. A useful and simple stand has a book-rest only, with an arrangement for altering the angle to suit the reader. The bed-tables, for holding larger things than books, *e.g.* the breakfast tray, can also be raised or lowered at will; the foot goes under the bed or sofa, and when not in use it forms a neat side-table. A very useful little present, in polished pine, stained walnut or mahogany, has four light legs which rest on the bed itself, to hold a book-rest, a cup of tea, etc. Back rests are made in great variety, and one we noticed was as comfortable as an armchair, very softly stuffed with hair, and with a cretonne covering.

The self-propelling "Merlin" and carrying chairs are always in demand; the latest production has hand rims fitted on to large rubber-tyred bicycle wheels for moving the chair easily and without noise, the back is adjustable to any angle, and if necessary the chair can be turned into an extended couch, with the leg rest raised; this makes a most comfortable reclining chair for an invalid, and the ease with which the patient's position can be changed without moving him off the chair makes the "Merlin" especially valuable to those who have to deal with persons recovering from illness. A strong and durable "Merlin" chair is made especially for hospitals.

Another exceedingly comfortable chair for all purposes has a shaped back; it is made in birch, oak, mahogany, or walnut, with the frame nicely carved and polished; it has a cane seat and back, a sliding foot rest and comfortable arm rests. The large bicycle wheels are fitted with rubber tyres and hand rim, and there is a smaller wheel behind.

Other invalid furniture may also be seen in large variety, including spinal carriages, bath chairs, etc., and there is a clever contrivan[illegible] for patients who have partially or entirely lost the use of t[illegible] lower limbs. This is a go-car or walking machine, and as it has only been perfected after many practical tests and long experience of the special requirements of such persons, it should supply a real need. In appearance it is rather like a tricycle, but the patient stands on the floor, with crutches under his arms, and these are fixed to the two large wheels; the height can be regulated at will, so that the same machine will fit tall, medium or short people. The small wheel goes first, and by holding to the horse-shoe shaped frame in front the lame man is able to walk.

It may be mentioned that invalid appliances, such as water and air beds, etc., can be had on hire from 12 Brompton Road. A passing notice must be given to the baby carriages, which are just as important from a hygienic point of view as carriages for older persons. Mr. Farmer has made a special study of the needs of the infant, and the result is a show-room specially devoted to baby carriages, mail carts, etc., many of which are very pretty. The "Royal Gig" is Mr. Farmer's own invention.

### BATHS AND SINKS FOR INSTITUTIONS.

(Doulton and Co., Ltd, Albert Embankment, London, S.W.)

A large variety of baths, sinks, etc., suitable for hospitals is on view at Messrs. Doulton's Lambeth Works; and in all, the object aimed at is perfect cleanliness combined with a pleasing appearance. Glazed ware is used wherever it is possible; it is strong, has a smooth surface not affected by use, is easily cleaned, and looks well. Everything is kept clear of the floor, so that cleaning is very much simplified: instead of standards cantilevers are used, and in all operating room fittings treadles for valves and waste are substituted for the old-fashioned plugs. This leaves the hands free, and prevents the fittings from being soiled. In all cases the ironwork is coated with vitreous enamel, which not only gives a smooth glazed surface, but prevents rust. For baths, iron is cheaper, lighter and more durable than porcelain, and it is more quickly heated to the temperature of the water. Messrs. Doulton now enamel their baths outside as well as in; thus there are no irregularities of surface to harbour dust or germs, and cleaning is very much simplified. The enamel is in several colours—"duck-egg" green, French grey or blue—as well as in white, to suit individual tastes. A contrivance worthy of notice is one for regulating the temperature of the water from either outside or inside the room. It is so arranged that cold water must be turned on first, so that the danger of scalding a helpless patient is lessened. The supply comes either through the bottom of the bath or over the top. In several designs the waste-pipe is exposed and removable, so that it may be brushed through with ease to prevent accumulation of dirt. It is also easy to get at the source of the mischief if the waste water does not run away. A special hospital bath is fitted with three indiarubber-tyred wheels; on the small front wheel there is a brake to keep the bath from running when in use.

The sinks for cleansing bed-pans and urine bottles are very thorough in their arrangements; they are provided with a rising spray and jet, one of which is worked by a treadle, and the other by a self-closing valve. The treadle is arranged so that as soon as the foot is removed the flushing cistern is discharged, thus the sink is automatically washed out immediately after use. One of these sinks has a brass-bound glass cover, made tight by means of a rubber joint, to prevent the danger of any offensive matter spreading beyond the rolled edge. The cover is hinged, and connected by a rod to the flushing cistern, so that each time the sink is used the cistern discharges, and an upward jet sprays into every corner. There are connections for hot and cold water, and the temperature can be regulated as needed. A steam jet for sterilising can be added to this sink.

A special lavatory is made for use in operating and dissecting rooms. Neither valves nor waste need be touched with the hands, being worked by treadle arrangements. Another has a constant flow of water by means of a spray.

Trouble frequently arises in water-closets from lack of a solid connection between the earthenware outlet and the soil-pipe; Messrs Doulton have patented a "Metallo-Keramic Joint," which is so finely soldered that the ware comes off in chips if any attempt is made to remove the lead. Any plumber can fix it.

Other hospital specialities shown are post-mortem tables and glazed ware stoves; the latter are in great variety and in artistic colours.

